# Libman-Sacks Endocarditis as the First Presentation of Systemic Lupus Erythematosus in a Young Man: A Multisystemic Diagnostic Challenge

**DOI:** 10.7759/cureus.117272

**Published:** 2026-09-30

**Authors:** Scott Waring, Joseph W Fletcher, Aaron Jones

**Affiliations:** 1 Acute Internal Medicine, Barking, Havering & Redbridge University Hospitals NHS Trust, London, GBR; 2 Acute Internal Medicine, Barts Health NHS Trust, London, GBR

**Keywords:** libman sacks endocarditis, neuropsychiatric sle, non-bacterial thrombotic endocarditis, sle (systemic lupus erythematosus), systemic lupus erythematosus

## Abstract

Systemic lupus erythematosus (SLE) is a complex, phenotypically varied disorder that possesses wide-ranging cardiac involvement. Libman-Sacks endocarditis (LSE) is one such cardiac manifestation, which results from the deposition of verrucous, sessile lesions favoring the left-sided heart valves. LSE is typically clinically silent without significant valvular dysfunction. Such patients with LSE, however, are those most susceptible to a more severe clinical phenotype resulting from SLE. Here, we report a unique case of a 22-year-old man initially presenting with unilateral leg swelling found to be secondary to a deep-vein thrombosis. He subsequently developed a constellation of acute symptoms, including embolic strokes, splenic infarction, proteinuric kidney injury, and significant mitral regurgitation, all of which mimicked bacterial endocarditis. His complex, multi-systemic disease course presented diagnostic challenges. We highlight the need in such complex cases for a specialist-led multidisciplinary approach, including cardiologists and rheumatologists, to guide serological and radiological investigations. Early use of echocardiography, in particular 3D transesophageal imaging, was paramount in clinching the diagnosis of LSE as an index presentation of SLE and in the transition from intravenous antibiotics to immunosuppression. Our case underscores the importance of the multidisciplinary team and the role of echocardiography in identifying such patients with LSE in SLE and thereby those most vulnerable to significant end-organ sequelae.

## Introduction

Systemic lupus erythematosus (SLE) is a complex, multi-systemic autoimmune disease with a chronic, relapsing-remitting course and highly variable multisystem manifestations that can range from mild to life-threatening [1]. Although its exact aetiology is not completely understood, the pathogenesis involves excessive production of damaging autoantibodies by B cells and cytokine dysregulation that can lead to end-organ damage.

Environmental factors, including sun exposure and smoking, as well as genetic predisposition, both contribute to these abnormal immune responses [2]. SLE is uncommon, with a worldwide prevalence estimated at 43.7 per 100,000 people, with women more frequently affected than men by a ratio of 9:1 [3].

Cardiac manifestations in SLE are diverse, including pericarditis, myocarditis, accelerated coronary disease and atherosclerosis, conductive disease and valvular involvement. Libman-Sacks endocarditis (LSE) is the characteristic valvular lesion of SLE; it is a form of non-bacterial thrombotic endocarditis (NBTE) characterised by sterile, fibrinous vegetations, and similar lesions also occur in primary antiphospholipid syndrome and malignancy [4]. LSE lesions are small (1-4 mm), verrucous lesions, which are sessile, favoring the left-sided mitral and aortic valves and most frequently the ventricular surface of the mitral valve, which may induce valve tip curling. Regurgitant dysfunction of the valve is therefore most common, but stenosis may also result from valvular thickening due to LSE lesion formation. Most patients with LSE, however, are asymptomatic, with valvular manifestations functionally mild, and the condition is often isolated incidentally [5]. Clinically overt valvular heart disease is uncommon, with regurgitation the predominant abnormality and stenosis rare, and only a minority of cases require valve surgery [6].

Valvular involvement is more frequent in patients with SLE who have antiphospholipid antibodies (aPL) than in those without [6], although valve lesions have also been described in aPL-negative patients. aPL are thought to promote thrombus formation on injured valvular endothelium, with the resulting fibrin-platelet thrombi organising into fibrosed, deformed valve tissue; immunoglobulin and complement deposition within affected valves has also been described, suggesting an additional immune-mediated mechanism. The presence of aPL appears to further increase the thromboembolic risk, mainly cerebrovascular, associated with valve lesions [6].

Despite a clinically silent course with regard to valvular disease, LSE is closely associated with neuropsychiatric SLE (NPSLE): this encompasses stroke, transient ischaemic attacks (TIA), confusional states, and seizures [7]. Patients with LSE have up to three times more circulating cerebromicroemboli, as well as reduced cerebral blood flow, increased stroke and TIA events, neurocognitive dysfunction, and greater brain lesion burden compared with those without LSE [7]. Overall, the presence of LSE is associated with a higher risk of embolic cerebrovascular disease and increased inpatient mortality in patients with SLE [8]. Timely detection of LSE is therefore essential, not only to differentiate it from infective endocarditis but also to identify a subset of SLE patients at greatest risk of morbidity and mortality who may benefit from immunomodulatory therapies and/or anticoagulation. We highlight LSE both as a diagnostic pitfall, mimicking infective endocarditis, and as a marker of a higher-risk SLE phenotype.

Here, we present a unique case of a previously well, young patient with an index presentation of SLE, complicated by LSE, who developed clinically significant valvular dysfunction and micro-embolic phenomena. Further disease complications with venous thrombosis, stroke, and lupus nephritis were also present, despite the lack of antiphospholipid antibodies.

This article was previously presented as a meeting abstract at the Lupus Annual Conference in Toronto, Canada (May 21-24, 2025).

## Case presentation

A 22-year-old man presented to the Urgent Treatment Centre (UTC) with symptoms of right lower limb swelling; he was otherwise well with no past medical history. A clinical suspicion of deep vein thrombosis (DVT) was established given findings of erythema, disproportionate swelling with comparison to the left lower limb, and an elevated D-dimer blood test of 7.34 mg/L (upper limit of normal: 0.5 mg/L). He was initiated on treatment-dose oral anticoagulation (rivaroxaban 15 mg per oral twice daily) and discharged with urgent outpatient Doppler ultrasound of the right lower limb to follow.

He returned to the Emergency Department nine days later with worsening right lower limb swelling despite compliance with treatment. Further history taking revealed the patient initially noticed his right lower limb swelling three months prior when he had travelled to the United Kingdom (UK) from his native country, and it had steadily progressed since then. More recently, he noticed left-sided pleuritic chest pain, in the absence of cough or shortness of breath. Neurologically, he also described isolated weakness in the left upper limb, causing him to drop items such as mugs. This weakness had been fluctuating over several weeks. He denied any right-sided upper limb, lower limb, or facial involvement. There was no speech disturbance reported or noted during consultation. On review of systems, he denied fevers but did report a two-week history of drenching night sweats.

By way of social history, the patient was of Southeast European origin, visiting family in the UK. He had previously worked in agriculture with both plants and animals. He was an ex-smoker with a 10-pack-year history and denied any recreational drug use or alcohol consumption. His only drug history was of the recently initiated rivaroxaban 15 mg twice daily, which he had been fully compliant with, and had induced minor self-resolving nosebleeds.

Positive findings on initial examination included an overtly swollen right lower limb, tenderness over the venous line, multiple splinter hemorrhages of both hands, and a soft pansystolic murmur loudest at the apex and on expiration. Lower limb examination noted an erythematous and swollen right calf, which was firm but non-tender, and> 3 cm in size compared to the left. Neurological examination noted slightly reduced left upper limb grip strength but was otherwise unremarkable, with normal power in all other regions, no sensory losses, normal tone and reflexes, and no cranial nerve signs. His respiratory exam was normal, and, importantly, there was no palpable cervical, supraclavicular, or axillary lymphadenopathy and no palpable hepatosplenomegaly. Regarding observations, he was febrile throughout his clinical course in the emergency department, with a peak temperature of 39.2 degrees Celsius (Table 1). 

**Table 1:** Observations upon presentation to the Emergency Department Br/min = Breaths per minute; RA = Room air; BPM = Beats per minute; EWS = Early warning score

Physiological parameter	Reading	EWS score
Respiratory Rate	28 Br/min	3
			Oxygen Saturation	98% RA	0
Blood Pressure (systolic/diastolic)	130/79	0			
			Heart Rate	91 BPM	1
Temperature	38.7 °C	1			
			EWS total	5	

Electrocardiogram (ECG) showed a sinus rhythm with voltage criteria of left ventricular hypertrophy, a narrow QRS with incomplete right bundle branch block morphology, and a low-normal PR interval (115 ms) with inferior T-wave inversion (Figure 1). Postero-anterior chest X-ray on arrival revealed a slightly enlarged cardiothoracic window, as well as blunting of both costophrenic angles, more so on the left than the right, and consistent small- to medium-volume pleural effusions (Figure 2). A computerised tomography (CT) examination of the head revealed small chronic infarcts in the left frontoparietal lobes, with no evidence of acute hemorrhage (Figure 3).

**Figure 1:**
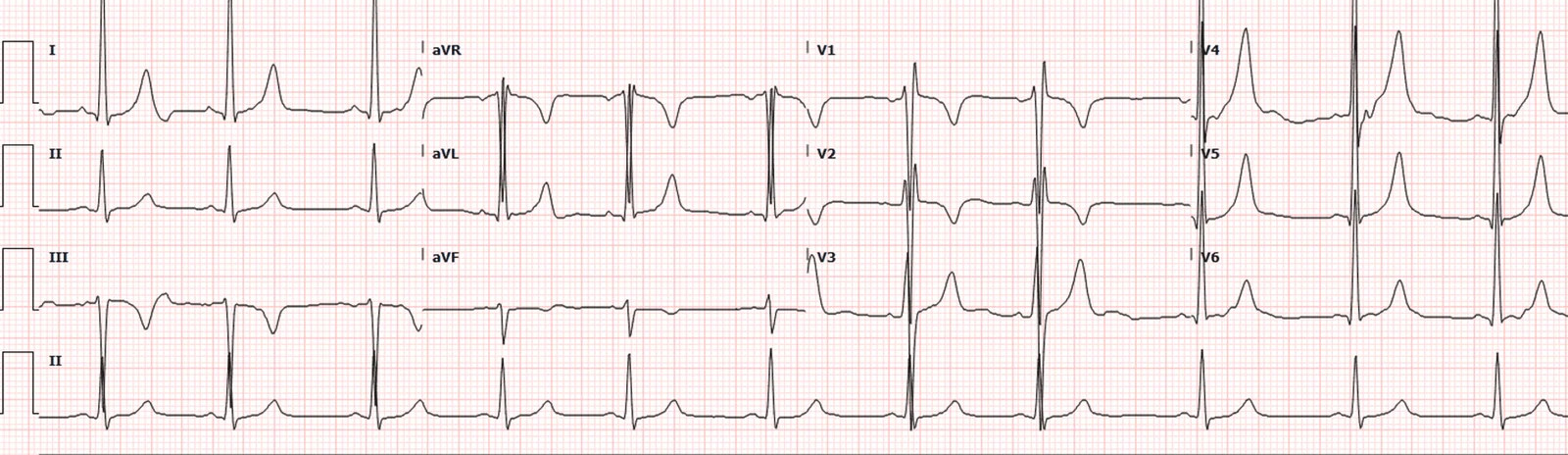
12-lead electrocardiogram from presentation to the Emergency Department, revealing a sinus rhythm with voltage criteria of left ventricular hypertrophy, a narrow QRS with incomplete right bundle branch block morphology, a borderline short PR interval, and inferior T-wave inversion

**Figure 2:**
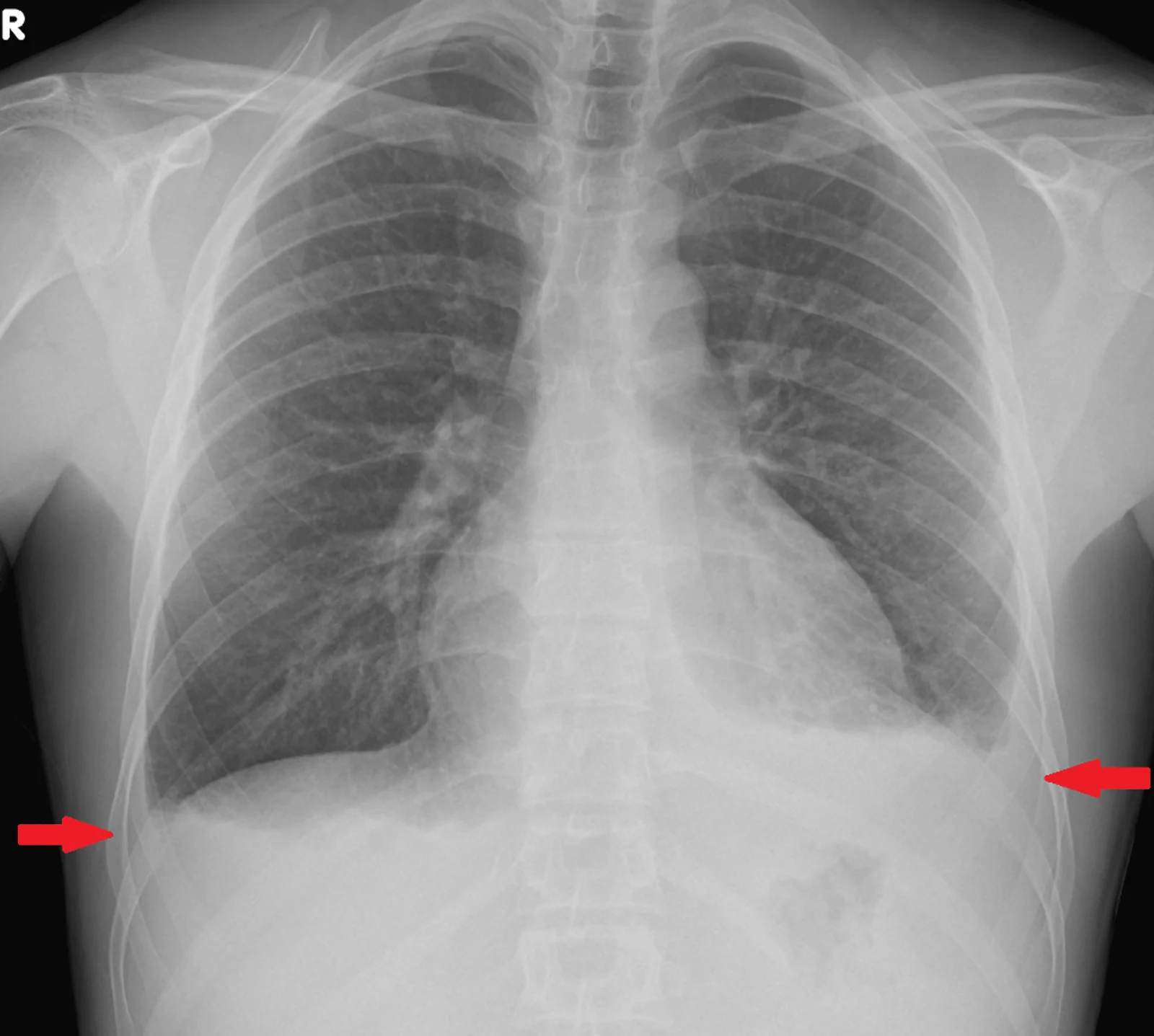
Chest X-ray in a postero-anterior view on presentation to the Emergency Department, revealing a mildly increased cardiothoracic ratio and bibasal pleural effusions (labelled)

**Figure 3:**
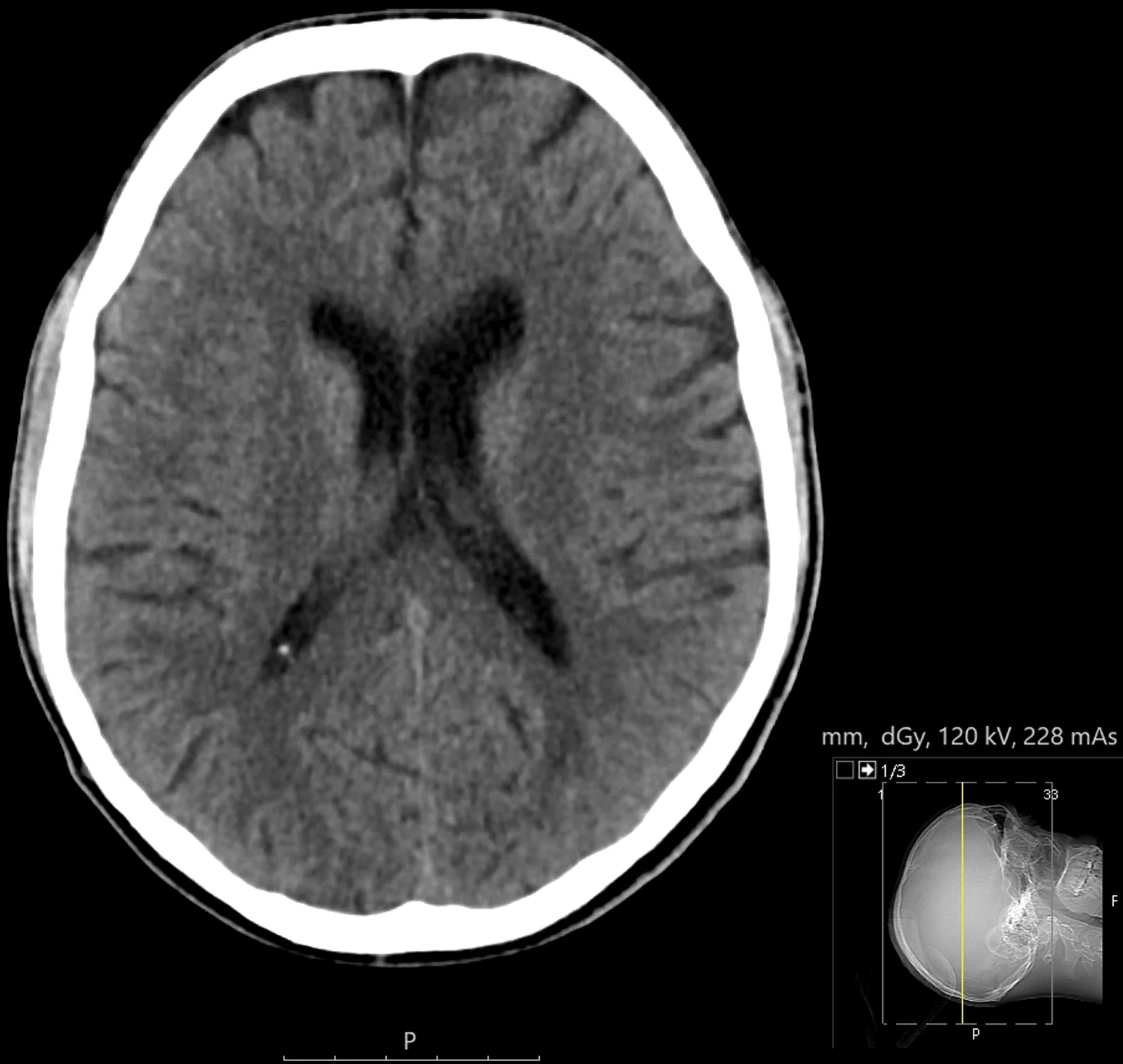
Computerised tomography imaging of the head in the axial plane revealing no significant pathological findings

Bloodwork revealed a normocytic anemia (hemoglobin (Hb): 101 g/L, mean corpuscular volume (MCV): 83 fL), mild thrombocytopenia (platelet count: 132 x 109/L), and mildly elevated C-reactive protein (13 mg/L). D-dimer was again elevated (3.61 mg/L), and clotting function was normal. Renal function revealed an acute kidney injury (creatinine: 141 µmol/L; previously 105 µmol/L at the time of initial presentation to the UTC; estimated glomerular filtration rate (eGFR): 61 mL/min/1.73 m^2^, previously 86 mL/min/1.73 m^2^) (Table 2). Liver function tests were normal. Venous blood gas revealed no evidence of acidosis (pH: 7.44; lactate: 0.5 mmol/L; HCO3-: 27 mmol/L). Routine admission retroviral screening was negative. Urine dipstick revealed 3+ blood and 3+ protein, with negative nitrites and leukocytes. 

**Table 2:** Bloodwork results for biochemical and serological screening on initial presentation to the Emergency Department eGFR: Estimated glomerular filtration rate; MCV: Mean corpuscular volume

Laboratory exam	Results	Reference range
Haemoglobin (g/L)	101	133-173
MCV (fL)	83	80-96
Platelets (10^9^/L)	132	150-400
Prothrombin time (seconds)	10.3	9-13
Activated partial thromboplastin time (seconds)	23	20-33
Fibrinogen (g/L)	2.68	1.5-4.0
D-dimer (mg/L)	7.34	0-0.5
Creatinine (µmol/L)	141	59-104
eGFR (mL/min/1.73m^2^)	61	>89
HIV 1&2 Ab p24 Ag	Not detected	-
Hepatitis C antibody	Not detected	-
Hepatitis B surface antigen	Not detected	-

A clinical suspicion for bacterial endocarditis was postulated, given the presence of an unexplained cardiac murmur, fevers, anemia, microscopic hematuria, embolic features with splinter hemorrhages, and presumed established embolic foci on CT of the head. Indeed, his Duke criteria for infective endocarditis were positive for three minor criteria at the point of admission. In view of this, appropriate investigations were instigated, including three sets of peripheral blood cultures being sent, and a transthoracic echocardiogram (TTE) was requested. The patient was empirically commenced on broad-spectrum antibiotics to cover for bacterial endocarditis (amoxicillin 1 g intravenous three times daily, and gentamicin 350 mg intravenous once daily as per body weight and creatinine clearance), aligning with local protocols.

An ultrasound Doppler scan of the right lower limb confirmed the presence of venous thromboembolism (Figure 4). A non-obstructive segmental acute thrombus of 43.2 mm length was noted in the common femoral vein, and occlusive acute superficial venous thrombosis in the long saphenous vein, extending from the saphenofemoral junction to the proximal calf. The left lower limb did not demonstrate venous thromboembolism. Subcutaneous oedema was also noted in the distal portion of both lower limbs. Following discussion with the hematology team, anti-cardiolipin and anti-phospholipid antibodies were sent to investigate for a cause such as antiphospholipid syndrome. The results of these tests were not available until later in the patient's admission and after further clinical deterioration. 

**Figure 4:**
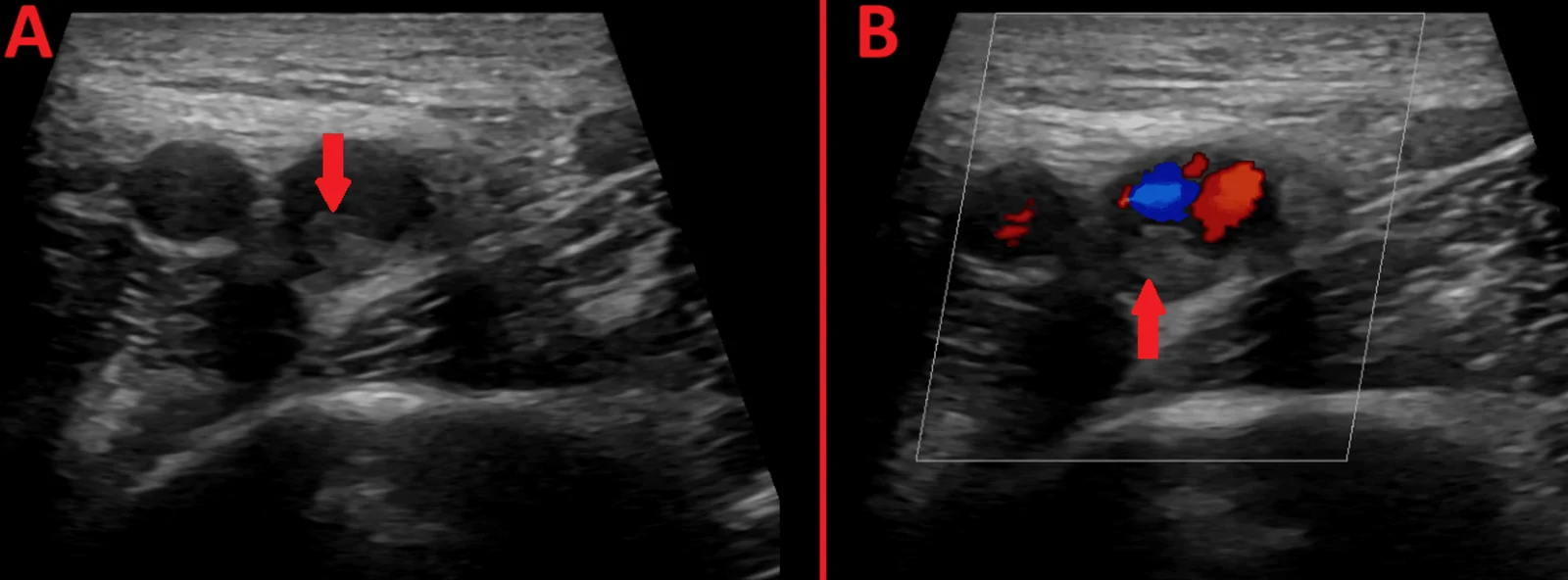
Ultrasound imaging of the right lower limb at the level of the common femoral vein revealing thrombosis (A), with Doppler flow confirming non-obstructive impairment of flow (B)

TTE revealed the mitral valve was abnormal; the anterior leaflet appeared thickened at the tip, while the posterior leaflet was shortened and mildly thickened, with moderate-to-severe mitral regurgitation with two regurgitant jets observed (Figure 5). There was no evidence of mitral stenosis. There was also a moderately dilated left ventricular cavity size with normal wall thickness and good function (left ventricular ejection fraction of 63%). Grade III diastolic dysfunction was present. Bi-atrial dilation and a dilated inferior vena cava (IVC) were also noted. The right ventricle and other heart valves were normal. A small global pericardial effusion was noted, with no evidence of hemodynamic compromise, at maximum depth 1 cm over the right ventricle. TTE was limited, however, in its inability to visualise any overt valvular lesions.

**Figure 5:**
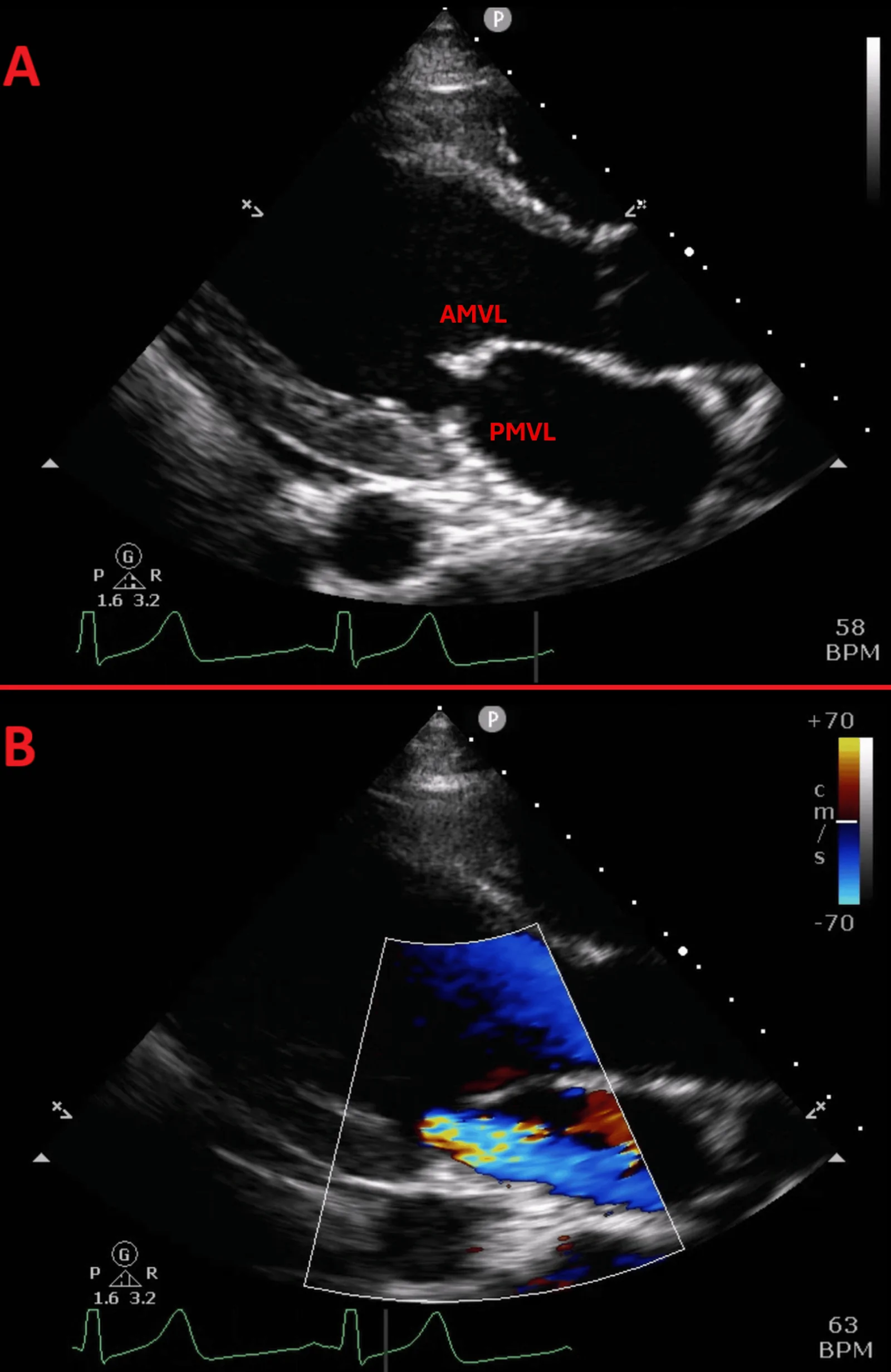
Transthoracic echocardiographic images in the parasternal long-axis view focused on the thickened anterior mitral valve leaflet and the shortened posterior mitral valve leaflet (pane A), resulting in significant mitral regurgitation on colour Doppler (pane B) AMVL: Anterior mitral valve leaflet; PMVL: Posterior mitral valve leaflet

In the presence of unexplained venous thromboembolism and pyrexia of unknown origin, further investigations were performed to isolate a unifying diagnosis. A CT scan of the thorax, abdomen, and pelvis was performed to exclude deep-seated infection or malignancy as a cause. This revealed a mildly enlarged spleen of 15 cm, with a peripheral wedge-shaped hypodensity suspicious for infarction (Figure 6). The liver was also slightly enlarged at 19 cm in long axis, with homogeneous parenchyma, and a normal biliary tree and common bile duct. The portal vein was patent. Other notable findings included the presence of multiple small-volume retroperitoneal and inguinal lymph nodes (< 1 cm). There was no thoracic lymphadenopathy nor notable thoracic findings. A subsequent testicular examination did not reveal any masses and was normal. As such, malignancy-related thrombosis was felt to be low in clinical probability, given the lack of clinical or radiological findings of underlying malignancy. 

**Figure 6:**
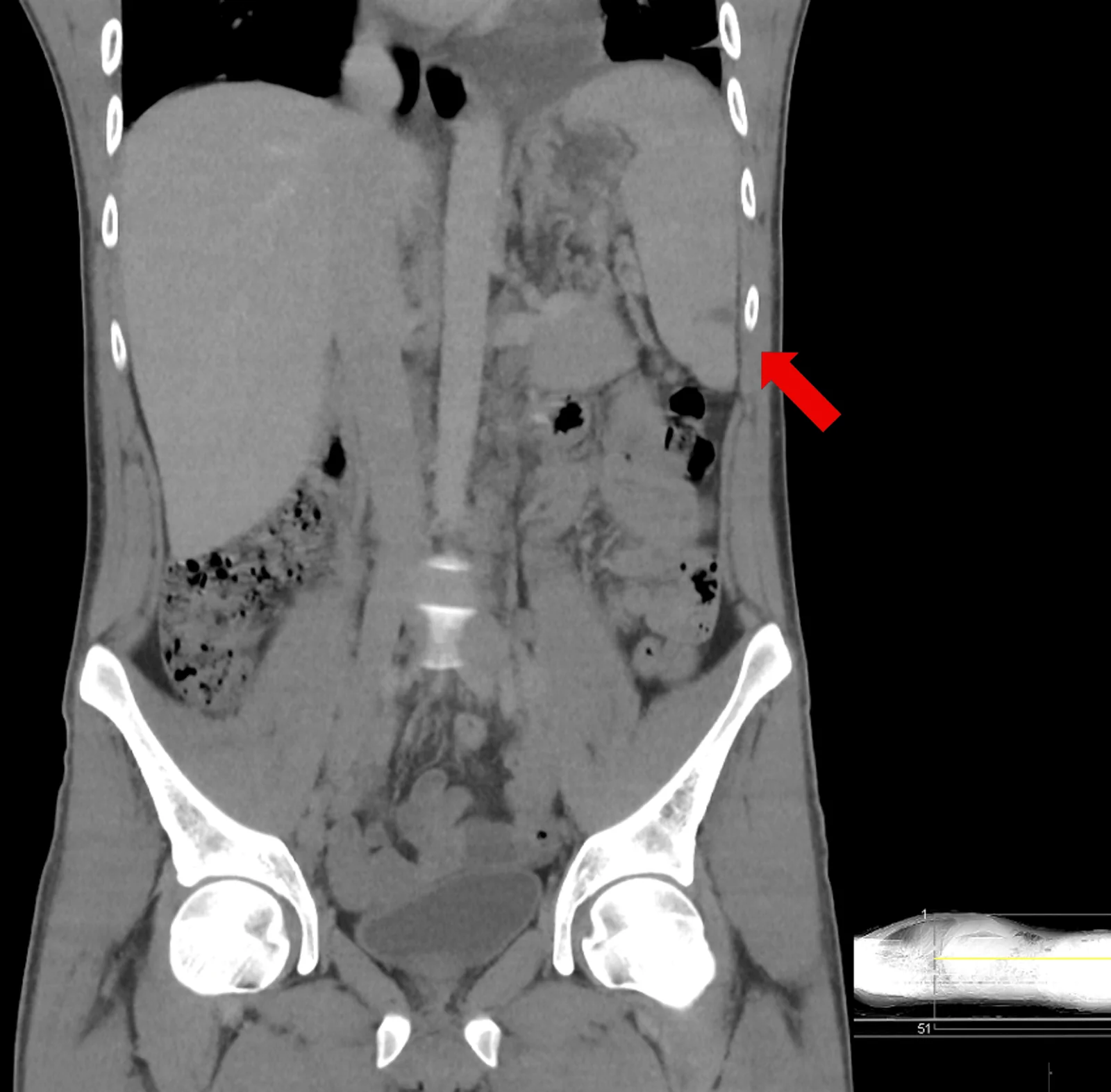
Computerised tomography coronal views revealing mild splenomegaly with a peripheral wedge-shaped hypodensity consistent with splenic infarction (labelled)

An MRI scan of the head was performed on Day 5 of admission in order to further characterize and evaluate the small chronic infarcts seen on initial CT imaging of the head. This revealed multiple small ischemic foci seen in the cortex of the parietal lobe bilaterally (Figure 7).

**Figure 7:**
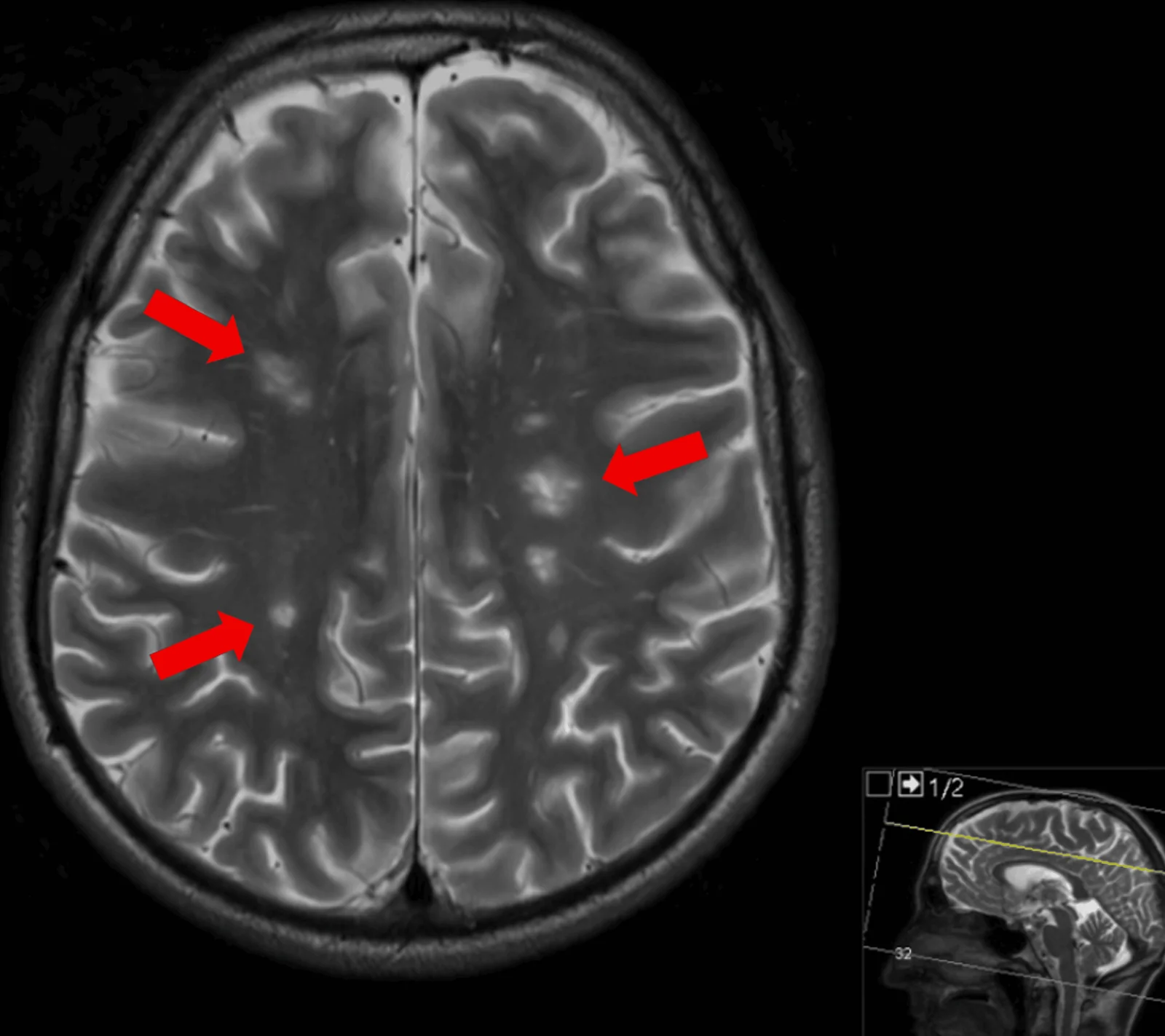
Magnetic resonance imaging of the head with axial views demonstrating scattered parietal lobe ischaemic foci (labelled)

Regarding initial blood culture findings, one anaerobic bottle tested positive for *Staphylococcus hominis*, felt to be a contaminant given the negativity of all other bottles. As such, sepsis-related embolic phenomena were felt to be unlikely. Treatment was maintained on broad-spectrum intravenous antibiotics, with daily ECG monitoring unchanged. Neurology was found to have recovered by Day 7 of admission, with a now entirely normal examination. The patient's antibiotic regimen was amended at this time with the addition of flucloxacillin 2 g intravenous four times daily, aligning with the tertiary cardiac center microbiological protocols for culture-negative endocarditis, prior to additional investigations and an agreement on definitive management planning. Culture-negative endocarditis serology was performed to exclude Q fever and brucellosis, particularly given the patient's exposure to livestock, which later returned negative.

The patient remained hemodynamically stable during this admission period, albeit with fluctuating low-grade temperatures throughout. A new lower limb rash was observed on Day 8 of admission, consistent with livedo reticularis. Regular inpatient blood tests revealed persistent normocytic anemia (Hb: 96 g/dL, MCV: 77 fL). Renal investigations found significant proteinuria with a microalbumin-to-creatinine ratio of 389 mg/mmol (normal limits: 0-30 mg/mmol), and the presence of red cell casts (>100 red cell count/uL on urinary microscopy). With nephrotic-range proteinuria (>3 g/24 hours), nephrotic syndrome was touted as a potential cause for this patient's venous thromboembolism; however, he did not meet criteria for this diagnosis owing to a lack of serum hypoalbuminaemia (albumin of 32 g/L). An autoimmune panel found complement levels were depleted (C3: 0.6 g/L, C4: 0.05 g/L). Anti-cardiolipin antibodies were negative (cardiolipin IgM antibodies: 8.2 kunits/L, normal limit: 0-10 kunits/L; cardiolipin IgG antibodies: 3.2 kunits/L, normal limit: 0-10 kunits/L). Anti-nuclear antibodies, however, were strongly positive (ANA antibody >31 IU/mL; ANA titer: >1:640; homogeneous pattern).

A transesophageal echocardiogram (TEE) was performed on Day 7 of admission to further characterize the patient's mitral valve disease, which was observed on TTE imaging, and to provide more valuable clinical information on the visual appearances and potential causes of this regurgitation. The images of this TEE were discussed at the local tertiary cardiothoracic centre endocarditis multidisciplinary team (MDT) meeting. TEE revealed findings of good left ventricular systolic function (LVEF). The mitral valve featured elongation and thickening of the anterior valve leaflet tip, again observed and better appreciated in the views obtained (Figure 8). Most notably, the mitral valve P3 leaflet was abnormal, with suspected perforation and a regurgitant jet seen at the base. Three-dimensional reconstruction of the images allowed the appreciation of multiple, small (1-2 mm), verrucous-type lesions, consistent with Libman-Sacks endocarditis (Figure 9). The overall impression was of moderate mitral regurgitation with no evidence of stenosis, and no urgent indications for cardiothoracic intervention. 

**Figure 8:**
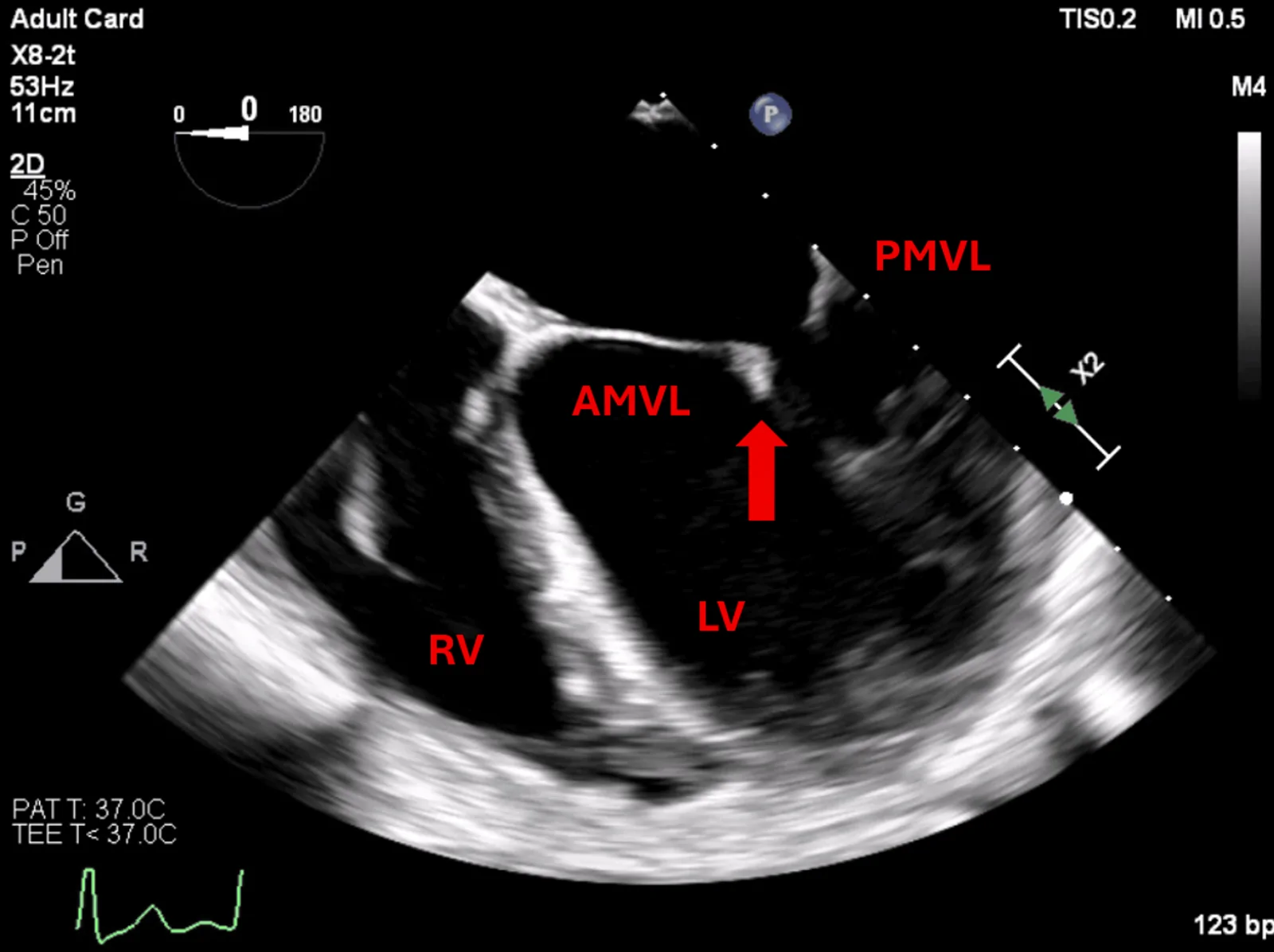
Transesophageal echocardiographic images revealing thickening and furling of the anterior mitral valve leaflet (labelled), with a shortening of the posterior leaflet LV: Left ventricle; RV: Right ventricle; AMVL: Anterior mitral valve leaflet; PMVL: Posterior mitral valve leaflet

**Figure 9:**
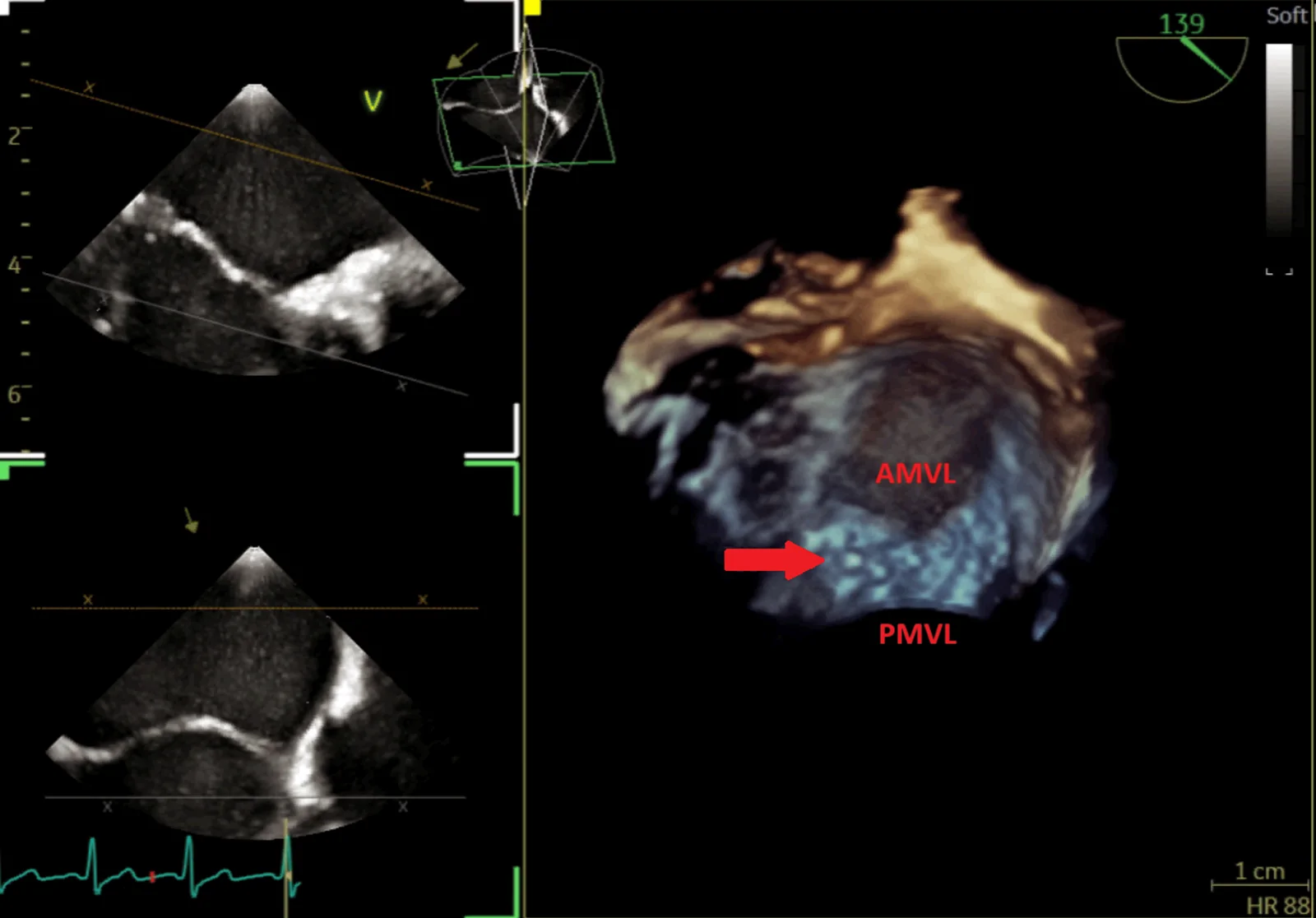
Three-dimensional transesophageal echocardiographic image reconstruction focused on the mitral valve revealing at least three small, verrucous lesions (labelled) attached to the posterior mitral valve leaflet, characteristic of Libman-Sacks endocarditis AMVL: Anterior mitral valve leaflet; PMVL: Posterior mitral valve leaflet

The patient was transferred to the tertiary cardiothoracic centre on Day 9 of admission to continue his in-patient management with specialist cardiology and rheumatology input. Multidisciplinary discussions with renal and rheumatology teams agreed upon a unifying diagnosis of systemic lupus erythematosus. This accounted for the positive auto-antibodies and depleted complement levels, and explained the patient's presentation with venous thromboembolism, livedo reticularis, cardiac and renal involvement (Libman-Sacks endocarditis and lupus nephritis, respectively), and multiple embolic features.

Understandably, debate within the MDT surrounded a discontinuation of antibiotics without certainty over the diagnosis. A renal biopsy was considered to confirm this diagnosis of SLE, but was agreed by the MDT to be too high risk given the need for concurrent anticoagulation for venous thromboembolism. Given persistent blood culture negativity and hemodynamic stability, the microbiologist agreed for the patient to commence high-dose oral steroids (prednisolone 60 mg per oral once daily) and discontinue intravenous antibiotics. With findings of declining renal function (at this time, his creatinine was 178 µmol/L; increased from 105 µmol/L on initial presentation to hospital; eGFR was now 42 mL/min/1.73 m^2^) with heavy proteinuria secondary to lupus nephritis, an angiotensin-converting enzyme inhibitor was also initiated on the advice of the nephrology team (ramipril 2.5 mg per oral once daily).

Serial TTE monitoring on Day 13 of admission showed preservation of LVEF (55-60%) with unchanged appearance of the mitral valve, including a thickened, bulky structure to the anterior mitral valve leaflet, contributing to moderate valvular regurgitation (Figure 10). A global pericardial effusion was again noted, at a maximum of 1.1 cm over the right ventricle, with no hemodynamic compromise. A repeat TEE on Day 18 once more found a thickened anterior mitral valve leaflet with sessile lesions on the posterior valve leaflet, particularly the distal tip (Figure 11). These were short and very bulky and felt to be compatible with Libman-Sacks endocarditis. The anterior mitral valve leaflet was free of lesions but was grossly thickened. Valvular regurgitation was graded to be mild with no evidence of perforation in this study, and other valves were overtly normal.

**Figure 10:**
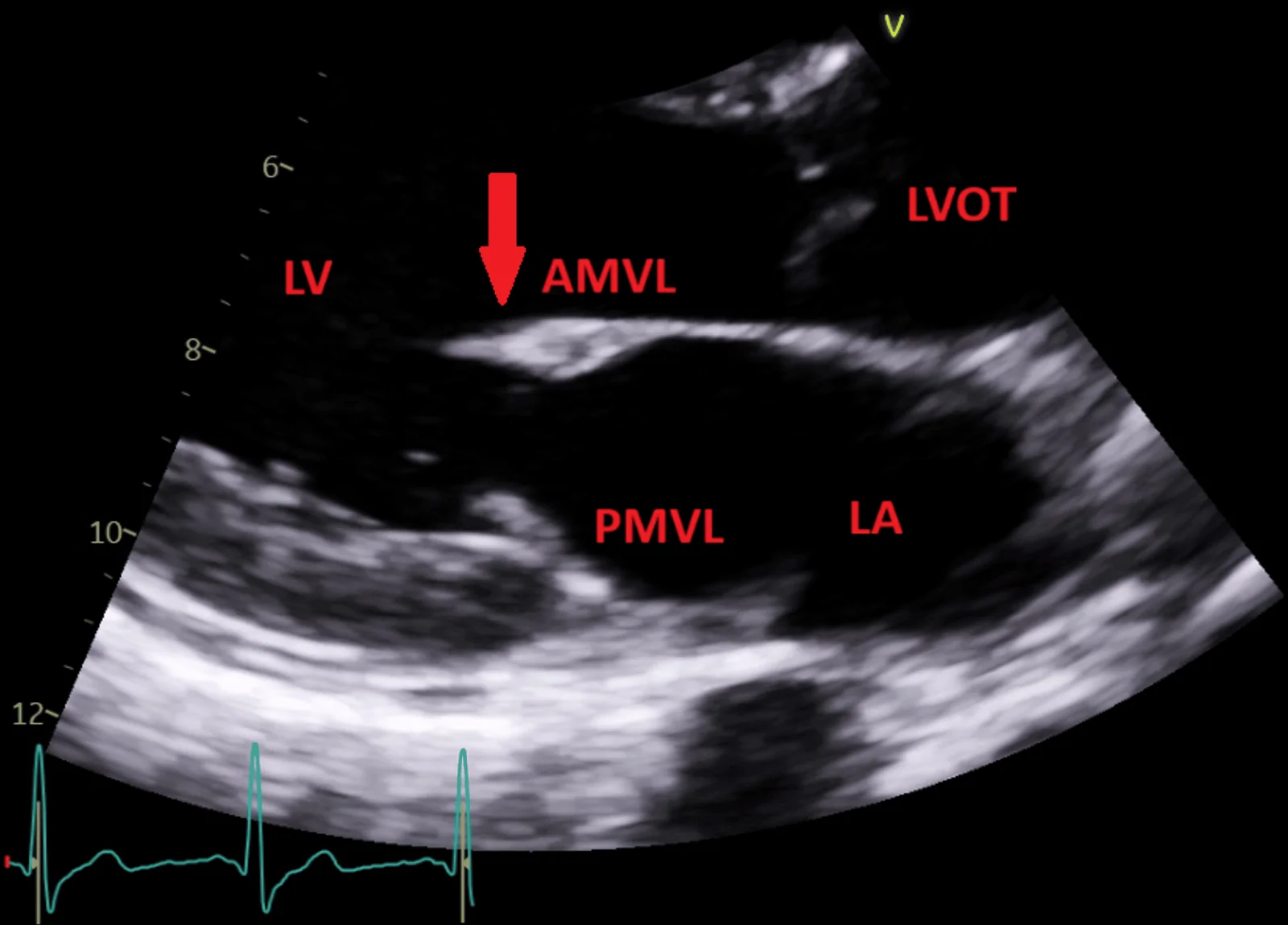
Transthoracic echocardiographic findings in the parasternal long axis view focused on the mitral valve, revealing a thickened anterior mitral valve leaflet tip (labelled) and a shortened posterior mitral valve LA: Left atrium; LV: Left ventricle; LVOT: Left ventricular outflow tract; AMVL: Anterior mitral valve leafle; PMVL: Posterior mitral valve leaflet

**Figure 11:**
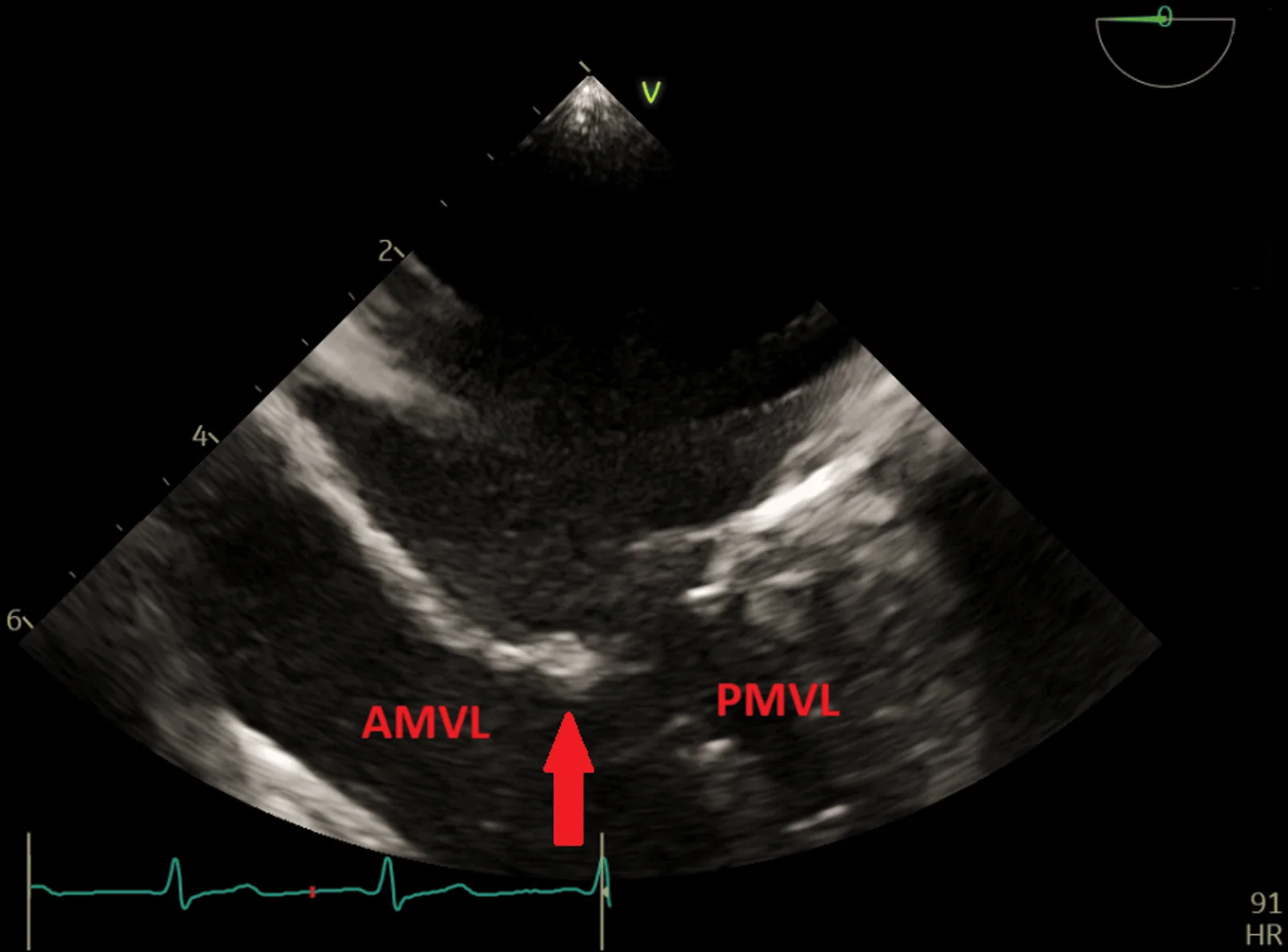
Transesophageal echocardiographic images focused on the mitral valve, revealing once more a bulky, thickened anterior mitral valve leaflet tip (labelled) and a shortened, hypomobile posterior mitral valve leaflet AMVL: Anterior mitral valve leaflet; PMVL: Posterior mitral valve leaflet

Extended auto-antibody testing was performed to confirm the diagnosis of SLE, but did not return until later into the patient's complicated admission. Within his connective tissue disease screen, as previously noted, anti-nuclear antibodies (ANA) were strongly positive, with a homogeneous pattern, and a dilution titre of >1:640. Anti-dsDNA antibodies were markedly elevated (332.0 k units/L; upper limit 10 kunits/L). Other antibodies, including proteinase 3 (PR3), myeloperoxidase (MPO), anti-cyclic citrullinated peptide (anti-CCP), anti-Jo-1, anti-scleroderma 70 (Scl-70), ribonucleoprotein (RNP), and anti-La antibodies. Anti-beta2 glycoprotein levels were negative (IgG: 4,000 units/L, IgM: 4,400 units/L). Cardiolipin antibodies were negative (IgG: 3,200 units/L, IgM: 8,200 units/L). Lupus anticoagulant antibodies were positive by dilute Russell's viper venom time (DRVVT) and dilute activated partial thromboplastin time (APTT), concordant with his SLE (Table 3). This extended connective tissue disease screen supported a diagnosis of SLE without the presence of antiphospholipid syndrome. 

**Table 3:** Bloodwork results for immunological screening

Laboratory exam	Results	Reference range
Complement C3 component (g/L)	0.6	0.90-1.80
Complement C4 component (g/L)	0.05	0.10-0.40
Cardiolipin IgM (kunits/L)	8.2	0-10
Cardiolipin IgG (kunits/L)	3.2	0-10
ANA antibody (mL)	>31	<1.0
ANA titre	>1:640	<1:80
Anti-dsDNA antibodies (kunits/L)	332.0	0-10
Proteinase 3 (PR3) antibody (kunits/L)	<0.2	<2.0
Myeloperoxidase (MPO) antibody (kunits/L)	<0.2	<3.5
Anti-CCP	Negative	-
Anti-Jo-1	Negative	-
Scl-70	Negative	-
RNP antibody	Negative	-
Anti-Ro (SSA)	Negative	-
La antibodies	Negative	-
Anti-beta2 glycoprotein IgG (kunits/L)	4.0	0-7
Anti-beta2 glycoprotein IgM (kunits/L)	4.4	0-7
Lupus anticoagulant screen (DRVVT)	Positive	-

The patient's renal function improved upon initiation of high-dose oral steroids (creatinine: 140 µmol/L on Day 25, from peak 178 µmol/L; eGFR: 55 mL/min/1.73 m^2^ down to 42 mL/min/1.73 m^2^). Amlodipine 10 mg per oral once daily was also initiated as an antihypertensive medication. At this point in time, with no indication for urgent cardiothoracic surgery and clinical stabilization achieved with high-dose steroids ongoing, the patient elected to self-discharge from the tertiary hospital in order to return to his native country for further inpatient monitoring.

## Discussion

LSE is a rare, characteristic, and under-recognized cardiac manifestation of SLE [5]. Valvular thickening and sterile verrucous vegetations are two commonly described forms of SLE-related valve involvement [9,10]. As illustrated by our case, the diagnosis of SLE and LSE can be difficult, requiring extensive investigations and numerous coordinated specialist opinions. Furthermore, distinguishing non-bacterial thrombotic endocarditis from bacterial endocarditis is challenging given the shared features of the two conditions [11]; indeed, patients with SLE and those on immunosuppressive medications will also be at greater susceptibility to bacterial endocarditis. It is clinically vital to differentiate the two conditions early to facilitate the successful management of these patients and initiate appropriate immunosuppressive medications, and this is perhaps also best appreciated by direct visualization of the valvular lesions themselves.

Evolving echocardiographic techniques have allowed a greater appreciation of characteristic LSE lesions [10], and here we demonstrated the additional diagnostic value of TEE compared to TTE alone. TTE often underestimates SLE-related valvular disease and is less reliable than TEE for differentiating LSE from infective endocarditis [9]. 3D-TEE possesses greater sensitivity and specificity than TTE for visualization of LSE vegetations. As seen in this case, LSE-related vegetations are sessile, typically smaller, are heterogeneous in echotexture, may show central calcification, and are visualized at the leaflet base. Conversely, bacterial lesions are homogeneous in echotexture and are found at the line of leaflet closure and move independently from the motion of the valve [10]. Given the importance of differentiation of the two appearances, a cardiologist-led multidisciplinary review of such images is therefore paramount.

Our case highlights a particularly unique presentation of a young man with clinically significant valvular dysfunction secondary to LSE, which contrasts with the typical silent and incidental findings of LSE in patients with SLE, often detected post-mortem or in long-term disease [5]. The patient had symptomatic mitral regurgitation, which was appreciated both on TTE and TEE, with findings of grade III diastolic dysfunction, bi-atrial dilation, and a dilated IVC. Our patient demonstrated typical features of nonbacterial thrombotic endocarditis (NBTE) with valvular thickening on initial TTE, and then on 3D-TEE imaging, characteristic multiple small, sessile lesions (Figure 9). The differentiation between the two is vital in enacting the correct treatment pathway, and it was the TEE imaging at the multidisciplinary team review that supported the decision for discontinuation of intravenous antibiotics in favor of high-dose steroids. The risk of exacerbating infection makes the switch to steroids in suspected endocarditis a difficult choice, and as such shared decision-making via a multidisciplinary approach including cardiologists and rheumatologists is advisable in such complex cases.

Autoimmune disease diagnosis is typically supported with biochemical testing of pathogenic connective tissue disease autoantibodies, such as in this case, which made the diagnosis of SLE evidently clear given strong positivity of anti-nuclear and anti-double-stranded DNA antibodies [12]. A pertinent difficulty with such antibodies when managing acutely unwell patients, however, is that their testing often takes time, particularly at a non-tertiary center with a dedicated laboratory on-site. As such, clinical diagnosis of SLE must be supported with other diagnostic modalities. In the case we have presented, the need for systemic anticoagulation with a confirmed DVT contraindicated the renal biopsy, which would traditionally be advisable in the setting of potential lupus nephritis. As such, diagnosis supported by 3D-TEE visualization of characteristic findings of LSE strongly supported the confirmation of SLE. Here, immunoglobulin screening showed the patient was positive for lupus anticoagulant (LA); other studies have demonstrated a correlation between positive LA and LSE [6,13]. Although LSE is most strongly associated with SLE in the presence of antiphospholipid antibodies, it has also been described in aPL-negative disease, albeit less frequently [6]. In this case, an early autoimmune screen (strongly positive ANA, hypocomplementaemia) supported the diagnosis of SLE independently of the valvular findings, and this, together with the TEE findings and clinical syndrome, informed the multidisciplinary decision to initiate immunosuppression ahead of confirmatory serology (anti-dsDNA, extended antibody panel), which became available later in the admission. This raises the question whether patients with confirmed LA and/or antiphospholipid syndrome with SLE coupled with specific risk factors (including historic thromboembolism, valvular disease, or neurological sequelae) might benefit from targeted TEE screening [14]. In turn, this may identify a subset of patients at greatest risk of an aggressive disease course.

As supported by our literature review, it is frequently observed that patients with LSE are at risk of NPSLE, relating to threefold greater numbers of circulating cerebro-microemboli per hour [7]. The proposed underlying mechanism is embolisation of platelet-fibrin thrombus from the valvular lesions into the cerebral circulation, with a possible additional contribution from immune-mediated endothelial injury [6,7]. This is clearly demonstrated in our case, whereby a 22-year-old man with limited risk factors presented with demonstrable upper limb weakness correlating with multiple small ischemic foci seen in the cortex of the parietal lobe bilaterally on MRI imaging (Figure 7). In such cases of strokes in young patients, autoimmune screening should therefore be a mainstay of diagnostic work-up to identify the causative etiology.

Here, our patient elected to self-discharge and return to his home country to continue his inpatient management. This limited our ability to monitor his response to immunosuppression and the evolution of his valvular disease and represents a limitation of this report; close early follow-up is otherwise advisable in such patients given the potential for progression of LSE and the need to titrate immunosuppressive therapy. Generally, cases of SLE with LSE are complex to manage, with few randomized trials to support the limited observational case studies. Treatment may include cardiothoracic surgery, with indications similar to those for bacterial endocarditis, such as heart failure, severe valvular dysfunction, large vegetations, recurrent embolization despite therapeutic anticoagulation, or acute valve rupture [15]. One study suggests that anti-inflammatory and anti-thrombotic therapy can reduce the number, diameter, and area of valve vegetations, thereby improving associated valve regurgitation and potentially avoiding the need for high-risk valve surgery [16]. Similar findings have also been reported in observational case studies [17]. This supports the view that treatment of the underlying autoimmune disease in SLE with immunomodulatory therapies entails the greatest outcomes for patients with multisystemic involvement.

## Conclusions

SLE is an autoimmune-mediated multisystem disease that can present with a wide range of symptoms of varying severities, presenting significant diagnostic and management challenges. These challenges can be compounded by the number of investigations and specialist input required, as well as the immediate management of an acutely unwell patient. There is often a delay in the availability of essential results required to confirm a diagnosis, such as autoantibody screening, and therefore timely commencement of the appropriate treatment. In this case, 3D-TEE findings characteristic of LSE directly informed the multidisciplinary decision to discontinue antibiotics in favour of high-dose immunosuppression ahead of serological confirmation. As such, as illustrated by our case, we advocate the use of other investigative modalities such as echocardiographic imaging to best appreciate the characteristic findings of LSE.

LSE is just one of many cardiac manifestations of SLE, but its presence is prognostically significant as it is associated with a more clinically severe phenotype and greater neuropsychiatric sequelae. In this case, we have demonstrated the importance of recognition of LSE given the multisystemic effects of this young patient's disease course, including lupus nephritis, venous thromboembolism, and embolic strokes. Here, we have revealed how shared decision-making is paramount to appreciating the overall diagnosis of non-bacterial thrombotic endocarditis in the context of SLE. Taking a multidisciplinary team-based approach with specialty review for each respective organ involvement allows for appreciation of a clinical diagnosis prior to formal confirmation through autoantibody screening, thereby expediting the timely management of such high-risk patients.
